# Can transformational leadership be associated with innovation among hotel employees? A dual examination of the mediating role of psychological empowerment and the moderating role of perceived organizational support

**DOI:** 10.3389/fpsyg.2026.1794461

**Published:** 2026-06-24

**Authors:** Ting Wan, Yong Miao

**Affiliations:** 1 Community Education College, Hubei Open University, Wuhan, Hubei, China; 2 Faculty of Continuing Education, Hubei University of Automotive Technology, Shiyan, Hubei, China

**Keywords:** hotel employees, innovative work behavior, perceived organizational support, psychological empowerment, transformational leadership

## Abstract

**Background:**

In the increasingly competitive hotel industry, innovation represents a central source of organizational advantage, and employees’ innovative work behavior forms the foundation of organizational renewal. Although prior research has explored the relationship between transformational leadership and employee innovation, less is known about how psychological empowerment and perceived organizational support operate together within hotel settings characterized by standardized service routines, emotional labor, and intensive customer contact. Grounded primarily in social cognitive theory and supplemented by leader-member exchange-informed relational reasoning, this study examined a moderated mediation model incorporating transformational leadership, psychological empowerment, innovative work behavior, and perceived organizational support.

**Methods:**

Standardized instruments were administered to 500 employees in Chinese hotel enterprises, and the data were analyzed using PLS-SEM.

**Results:**

Transformational leadership was positively related to innovative work behavior, and psychological empowerment partially accounted for this relationship. Perceived organizational support positively moderated both the transformational leadership–innovative work behavior link and the psychological empowerment–innovative work behavior link, with a stronger moderating pattern for the former. Multi-group analysis further showed that the relationship between transformational leadership and psychological empowerment was stronger among female employees.

**Conclusion:**

The findings highlight the joint relationships among transformational leadership, psychological empowerment, perceived organizational support, and innovative work behavior among hotel employees. The study provides theoretical and practical insights for leadership practices and innovation in hotel settings.

## 1 Introduction

In today’s rapidly changing hotel sector, sustaining growth and gaining a competitive edge depend largely on continuous innovation. Within organizations, such innovation is primarily rooted in employees’ innovative work behavior (IWB), which refers to a set of work-related behaviors through which individuals generate, promote or champion, and implement novel and useful ideas in their professional roles (De Jong and Den Hartog, 2010; Scott and Bruce, 1994). In hotel settings, IWB is reflected in employees’ efforts related to service process refinement, customer-experience optimization, and operational-practice adjustment through idea generation, idea advocacy, and idea implementation (Lee and Shin, 2025). However, hotel innovation unfolds under conditions that differ from many other organizational contexts. Frontline service employees often work within standardized service routines while facing heterogeneous customer requests, emotional labor demands, time pressure, and intensive face-to-face interactions (Lam and Cheung, 2024; Xiong et al., 2023). In service encounters, frontline employees may also need to adjust or improvise around standardized procedures when unpredictable customer requests arise (Rayburn et al., 2025). These features mean that innovative suggestions may be valuable but also risky, because employees must balance service consistency with situated adaptation. Conversely, a lack of innovation may be accompanied by organizational rigidity and reduced adaptability to market changes. Beyond individual factors such as creative self-efficacy (Zhuoma et al., 2025) and personality traits (Stergiopoulou and Meurs, 2025), innovation also emerges from the dynamic interaction between individuals and their environments.

According to social cognitive theory (SCT) (Cervone, 2023), individual behaviors such as IWB result from the continuous interaction between personal cognitive factors and environmental influences. This perspective highlights that research should not focus solely on individual traits but also consider the interplay between external contexts and internal psychological mechanisms. Within organizational settings, leadership style is a key external factor. Transformational leadership (TL), characterized by idealized influence, intellectual stimulation, and individualized consideration, motivates employees to challenge conventions and supports their innovative efforts. TL is associated with stronger innovation intentions and is linked to a psychologically safe climate grounded in trust and support, which is related to employees’ engagement in innovation through reciprocity (Karimi et al., 2023; Korku and Kaya, 2023). Furthermore, leader-member exchange theory (LMX) posits that high-quality leader-member relationships provide employees with resources, trust, and autonomy, making them more likely to go beyond formal role expectations and engage in extra-role behaviors such as innovation (Jelaca et al., 2025).

From a cognitive perspective, psychological empowerment (PE) constitutes an essential cognitive resource that fosters innovation. Employees who view their work as purposeful, possess a sense of competence, feel autonomous, and believe in their capacity to affect outcomes tend to recognize challenges, seek alternative solutions, and engage more actively in realizing creative ideas (Vu et al., 2025). Empirical studies have shown that individuals with elevated PE exhibit greater participation in IWB (Bektas et al., 2025; Stanescu et al., 2021). Moreover, perceived organizational support (POS) functions as a contextual enabler that strengthens these dynamics. When employees perceive their organization as valuing their contributions and providing support, their intrinsic motivation is more easily translated into creative and innovative efforts (Lin et al., 2024).

Although prior research has accumulated substantial evidence linking TL with employee creativity and IWB, this study does not position its contribution in the general TL–innovation association itself. Recent hospitality research has already examined TL and IWB in relation to organizational identification, employee voice, and innovation climate (Lin, 2023). However, less is known about how hotel employees’ PE and POS operate together within one SCT-based moderated mediation framework, especially when POS is treated as a broader organizational support boundary rather than another leadership-related construct. Drawing primarily on SCT, and using LMX only as a supplementary relational lens, this study traces how TL is associated with IWB, tests the mediating role of PE, and assesses whether POS is associated with variation in these links. This narrower framing clarifies the specific gap addressed in this study: not whether TL is generally related to innovation, but under what perceived organizational support conditions leadership-related cues and empowerment-related cognitions correspond to hotel employees’ IWB.

## 2 Theory and hypotheses

### 2.1 Theoretical foundation

This study primarily draws on SCT to form an environment–cognition–behavior framework. SCT posits that behavior emerges from dynamic interactions among environmental conditions, individual cognitions, and behavioral responses (Bandura, 2001). In this study, TL and POS represent environmental factors, PE reflects a key cognitive factor, and employees’ IWB constitutes the behavioral outcome (Kim and Yoon, 2025). This framework provides the basic logic for understanding how external contexts relate to internal cognition and, in turn, to specific behavioral expressions (Peng and Chen, 2025). LMX is used as a supplementary relational perspective rather than as a measured mediator or moderator (Graen and Uhl-Bien, 1995). Because LMX is not included as a latent construct in the empirical model, this study does not treat LMX as an explanatory mechanism tested by the data. Instead, LMX-informed reasoning helps clarify why employees may interpret TL behaviors, such as individualized consideration and intellectual stimulation, as proximal relational cues. The empirical model itself focuses on the observed associations among TL, PE, POS, and IWB.

Taken together, SCT provides the main theoretical chain from environment to cognition and then to behavior, whereas LMX offers a supporting lens for interpreting the relational meaning of TL behaviors. In addition, this study treats POS as a critical contextual variable and examines its moderating role in the strength of the aforementioned associations.

### 2.2 Transformational leadership and innovative work behavior

TL is a leadership style that promotes organizational change and innovation by setting high performance expectations, stimulating higher-order intrinsic motivation among subordinates, and shaping a supportive organizational climate (Bass, 1999). In the hotel industry, where human capital and service quality are central, TL emphasizes idealized influence, inspirational motivation, intellectual stimulation, and individualized consideration, and it shows a direct association with employees’ IWB (Sürücü et al., 2021).

A large body of research shows a close association between TL and employees’ IWB. Korku and Kaya (2023) report that TL encourages employees to question routines and provides a sense of safety and support for trying new approaches, which relates to stronger innovation intentions and proactivity. Bak et al. (2022) further note that, unlike leadership styles centered on transactional exchange, TL emphasizes the activation of followers’ work motivation and value identification, which predicts performance that goes beyond formal role requirements; IWB is a prototypical form of such extra-role behavior. Based on LMX, leaders and subordinates develop relationships that vary in quality (Graen and Uhl-Bien, 1995). Through individualized consideration and trustful support, TL is associated with higher-quality relationships, which relate to greater access to resources, trust, and autonomy. Evidence from Karimi et al. (2023) suggests that this positive and reliable relational context provides an ideal setting for innovation, and employees, guided by reciprocity norms, are more willing to respond with proactive innovation to leadership and organizational support. Nevertheless, the accumulated evidence should not be read as indicating a uniform TL–IWB association across all hotel settings. Prior hospitality research has already examined the TL–IWB relationship through organizational identification, employee voice, and innovation climate (Lin, 2023). These constructs refer to different theoretical domains: innovation climate captures whether new ideas are collectively supported, organizational identification reflects perceived oneness with the organization, and employee voice refers to the expression of constructive suggestions. POS is theoretically distinct from these constructs because it captures employees’ generalized belief that the organization values their contributions and cares about their well-being. Therefore, POS represents a broader organizational support signal rather than a specific innovation climate, identification process, or voice behavior. Therefore, we propose that TL is positively associated with IWB.

Moreover, the association between TL and innovation does not operate only through a direct path. According to SCT, the effect of environmental factors on behavior is often realized through individuals’ cognitive states (Kim and Yoon, 2025). PE functions as a key internal cognitive link in this mechanism. Prior studies indicate that TL is associated with higher PE. Raza and Yousufi (2023) report that vision articulation and individualized consideration relate to stronger perceptions of work meaningfulness, autonomy, and competence. Empirical results from Cekmecelioglu et al. (2023) also support a positive association between TL and PE. At the same time, PE itself relates positively to IWB. For example, Al Daboub et al. (2024) find that PE strengthens proactive exploration, problem identification, and solution implementation. When employees perceive their work as meaningful and feel capable and influential, they tend to generate and implement new ideas. Furthermore, Vu et al. (2025) provide direct mediation evidence showing that the positive association between TL and IWB is partly transmitted through PE.

Based on this theory and evidence, we further examine the internal pathway linking TL to hotel employees’ IWB and test whether PE mediates this association.

### 2.3 Mediating role of psychological empowerment

In organizational behavior and psychology, PE refers to a multifaceted construct grounded in intrinsic motivation that captures employees’ positive, task focused evaluations of their work roles. It comprises four core facets: meaning, competence, self-determination, and impact. Taken together, these facets operate as an integrated profile that represents an individual’s overall level of empowerment (Li J. et al., 2025). In practice, empowered employees endorse the value of work goals, feel confident in their capabilities, experience autonomy in role execution, and believe they can influence work systems and outcomes.

Grounded in SCT, behavior emerges from ongoing interactions between cognitive and environmental factors. In hotel settings, TL represents a key environmental factor. For its influence to translate into sustained innovative actions, employees’ internal cognitive states often serve as the conduit. Empirical studies show that TL, through vision articulation and individualized support, is associated with higher PE, and greater PE relates positively to innovative behavior (Bektas et al., 2025). This evidence suggests that leadership behavior can first empower employees’ cognitions, encouraging them to identify problems, generate solutions, and advance implementation because they perceive value and autonomy in their work, thereby turning leadership’s innovation advocacy into concrete action. A supplementary relational perspective is also useful for interpreting this pathway. Although LMX is not measured in the present model, LMX-informed reasoning suggests that employees may read individualized consideration and developmental support as relational cues associated with trust, access to resources, and autonomy (Peng and Chen, 2025). These relational cues are conceptually relevant to the meaning, competence, self-determination, and impact dimensions of PE (Peng and Chen, 2025; Stanescu et al., 2021). However, the empirical test in this study concerns the TL–PE–IWB associations rather than an LMX-mediated pathway.

In addition, prior work provides direct support for this mediating route. Vu et al. (2025) identified PE as a mediator linking TL with employees’ IWB. Consistent with these findings, Stanescu et al. (2021) reported that PE was positively linked to IWB and noted that the joint motivational force of meaning, competence, and autonomy provides a psychological basis for translating innovative intentions into action.

Taken together, TL may show an indirect link to IWB through elevated PE. Accordingly, we propose that PE serves as a mediator between TL and IWB among hotel employees.

### 2.4 Moderating role of perceived organizational support

POS reflects employees’ belief that their organization appreciates their efforts and is genuinely concerned with their overall welfare, encompassing both emotional recognition and the provision of tangible resources. This construct needs to be distinguished from both TL and LMX. TL refers to employees’ perceptions of supervisors’ transformational behaviors, whereas POS refers to employees’ generalized evaluation of the organization as a whole. LMX, by contrast, concerns dyadic exchange quality between a leader and a subordinate, but it is not measured in the present model. Thus, POS is modeled as a broader organizational context that may condition the TL–PE–IWB associations, rather than as a substitute for leadership behavior or leader–member exchange. In hotel settings where high pressure coexists with demanding service standards, POS functions as a key social-context resource that is associated with emotional recognition, perceived safety, and access to tangible support, thereby laying a psychological foundation for innovation-related behavior (Cheng et al., 2026).

Grounded in SCT, behavioral outcomes arise from the interaction of environmental, cognitive, and individual factors (Ye et al., 2025). As an external contextual variable, POS shapes how employees cognitively process leadership behaviors and their own capabilities. When employees perceive greater trust and support, they tend to interpret the empowering signals of TL as genuine and credible, which relates to higher PE; when perceived support is low, this motivational effect is likely to weaken (Ergun et al., 2025; Yildirim and Naktiyok, 2017). Accordingly, POS may reinforce the association between TL and PE. It may also be relevant to how PE is translated into innovative practice. PE is associated with stronger innovation intentions, yet the transition from intention to action typically requires a safe, inclusive, and well-resourced organizational climate. High levels of POS help translate empowerment-driven intrinsic motivation into visible IWB, encouraging employees to accept trial-and-error risks and adopt new methods. Insufficient support may inhibit this conversion (Al-Taie and Khattak, 2024). In addition, POS may shape the direct association between TL and IWB. Under high-support conditions, employees receive greater recognition and resources, and leaders’ vision and encouragement are more likely to externalize into concrete innovative practices. Under low-support conditions, resource constraints or fear of failure may reduce employees’ innovation-related engagement (Ergun et al., 2025). Therefore, we expect POS to moderate the mediation model that includes TL, PE, and IWB, thereby influencing the strength and, potentially, the direction of the associated relationships.

### 2.5 The present study

In the hotel industry, innovation manifests not only in service process optimization, customer experience enhancement, and digital technology integration but also in employees’ continuous engagement in identifying problems, proposing improvements, and implementing new ideas. Given the industry’s high level of interpersonal interaction, emotional labor, and dependence on human capital, employees’ IWB directly affects service quality, customer satisfaction, and operational efficiency. Accordingly, innovation carried out by employees forms the foundation of organizational innovation.

Although earlier studies have revealed a direct connection between TL and innovation and discussed the mediating function of PE, recent hospitality evidence indicates that the TL–IWB relationship has also been examined through organizational identification, employee voice, and innovation climate (Lin, 2023). Therefore, the present study does not claim that the TL–innovation link itself is unexplored. Instead, two more specific gaps remain: (1) the joint placement of PE and POS within one hotel-specific moderated mediation framework remains insufficiently specified; and (2) it remains unclear under what perceived organizational support conditions leadership-related cues and empowerment-related cognitions show stronger or weaker associations with hotel employees’ IWB.

To address these gaps, this study advances theory in two key ways. First, it refines rather than originates the TL–innovation discussion by examining it within the distinctive hotel context, where standardized service routines, emotional labor, and intensive customer contact shape employees’ room for innovation-related behavior. Drawing primarily on SCT, we develop an “environment–cognition–behavior” model encompassing leadership behavior, employee cognition, and organizational support, offering a systematic explanation of how TL is associated with innovation among hotel employees. Second, this study specifies the organizational boundaries of these associations by introducing POS as a critical contextual variable to delineate the perceived support conditions under which TL–PE–IWB associations become stronger or weaker. This approach does not ask simply whether TL is related to innovation, but instead addresses when leadership-related cues and empowerment-related cognitions correspond more closely to hotel employees’ IWB, thereby enhancing the alignment between theoretical depth and managerial relevance.

**Figure 1:**
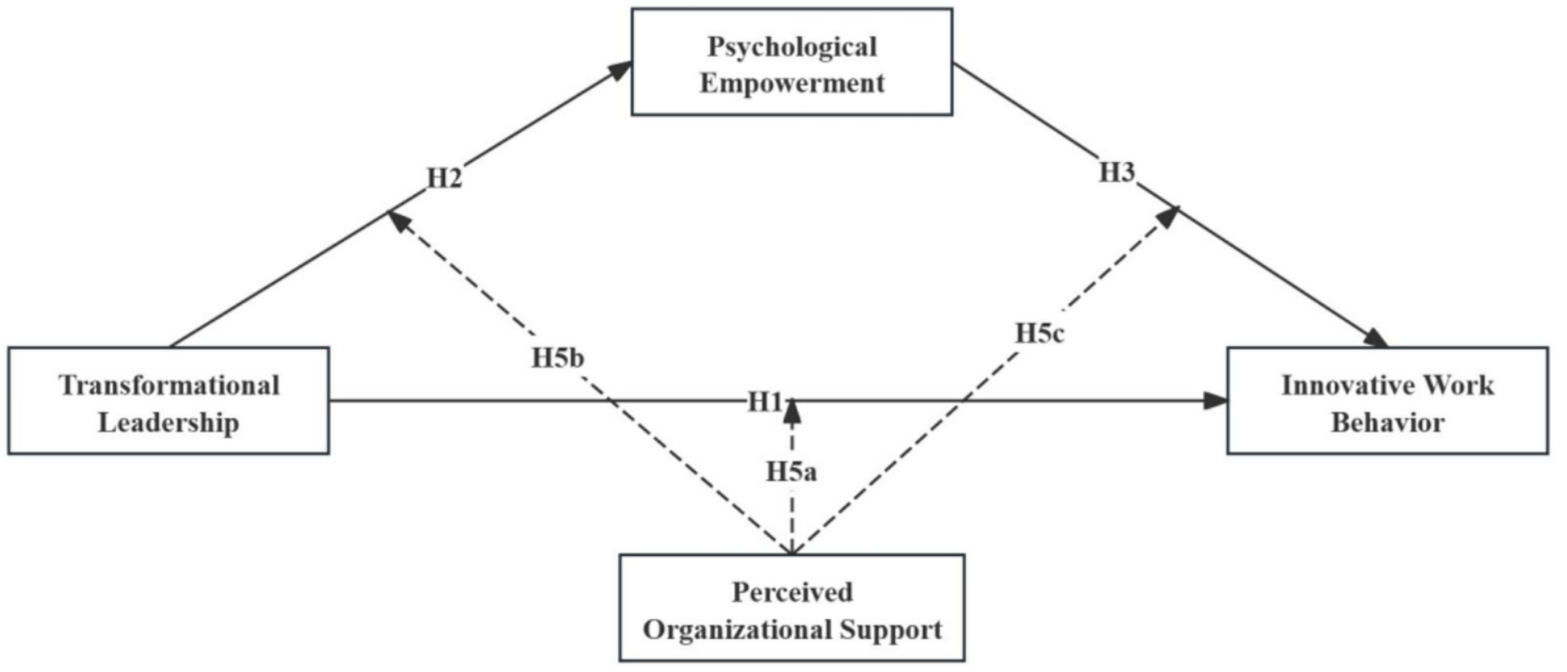
Theoretical model and hypotheses.

Consequently, the present research seeks to clarify both the underlying process and contextual conditions linking TL to hotel employees’ IWB by employing a moderated mediation framework. Drawing upon prior theoretical insights and empirical findings, we develop the conceptual framework shown in Figure 1 and articulate the subsequent hypotheses.

*H1*: TL is positively associated with IWB.

*H2*: TL is positively associated with PE.

*H3*: PE is positively associated with IWB.

*H4*: PE mediates the association between TL and IWB.

*H5a*: POS positively moderates the direct association between TL and IWB.

*H5b*: POS positively moderates the association between TL and PE.

*H5c*: POS positively moderates the association between PE and IWB.

## 3 Materials and methods

### 3.1 Participants

This cross-sectional study surveyed employees from hotel enterprises across China. We used a multi-stage sampling procedure that combined hotel-level recruitment with random employee selection within participating hotels. In the first stage, hotels were recruited through an industry database and partner contacts from multiple regions of China, including North China, East China, and South China, with varied hotel categories and operational settings. In the second stage, after management approval had been obtained at each participating hotel, complete employee rosters were assembled as within-hotel sampling frames. Computer-generated random numbers were then used to select eligible employees from each roster. Under this procedure, each eligible employee within the participating hotel rosters had a known chance of selection.

To support generalizability, hotel administrators briefed randomly selected employees on the study purpose, relevance, and confidentiality safeguards, encouraging voluntary participation. Participants filled out the questionnaire independently and by choice. Before starting the survey, participants were informed that there were no right or wrong answers, that their responses would be used only for research purposes, and that individual responses would remain confidential. The questionnaire retained the original scale blocks and item wording from the validated instruments rather than using item counterbalancing; all five-point Likert-type response options were displayed with clear endpoint anchors from 1 (strongly disagree) to 5 (strongly agree). Data collection took place anonymously on the Sojump platform (www.Sojump.com) between September and November 2025. Following common recommendations for sample size estimation, using a 10:1 respondent-to-item rule, the 38-item questionnaire and a 20% attrition allowance yielded a target sample of 456 (Kline, 2023). We distributed and received 530 questionnaires. We excluded invalid cases using three rules: (1) more than five missing items, meaning less than 80% completion; (2) highly uniform responding, defined as identical ratings on more than 80% of items such as “strongly agree” or “strongly disagree”; and (3) insufficient effort, defined as a completion time shorter than one third of the median survey duration (Wu et al., 2026). Applying the screening rules yielded 500 valid questionnaires, with valid responses accounting for 94.34%. The final dataset included 500 respondents, with 247 men and 253 women. These respondents came from 18 participating hotels located in North China, East China, and South China. The participating hotels covered diverse operational settings, including midscale, upscale, and luxury hotels, and included both chain-affiliated and independent hotels. Specifically, 12 hotels were chain-affiliated and 6 were independently operated. This distribution provided coverage of hotels with different regional locations, service levels, and ownership structures. Table 1 summarizes the demographic characteristics.

**Table 1:** Participant demographics (*N* = 500).

Demographic characteristics	Category	Quantity	Percentage (%)
Gender	Male	247	49.4
	Female	253	50.6
Age	18–25	81	16.2
	26–30	106	21.2
	31–35	116	23.2
	36–40	101	20.2
	>41	96	19.2
Length of service	<1	105	21.0
	2–4	119	23.8
	5–7	110	22.0
	8–10	89	17.8
	>11	77	15.4

### 3.2 Measures

#### 3.2.1 Transformational leadership

We assessed TL with the scale by Dai et al. (2013). The instrument contains 8 items that capture employees’ perceptions of supervisors’ transformational behaviors, including motivational and supportive actions. One item states, “My leader understands my situation and offers encouragement and help.” Respondents used a five-point Likert scale from 1 (strongly disagree) to 5 (strongly agree). Higher ratings indicate stronger perceptions of transformational leadership. Evidence from Chinese organizational settings supports the scale’s psychometric quality (Jiatong et al., 2022). In the final analysis, TL showed a Cronbach’s *α* of 0.937.

#### 3.2.2 Psychological empowerment

We evaluated PE with the instrument developed by Spreitzer (1995). The 12-item measure spans four domains: meaning, competence, self-determination, and impact. Example items include “Ton a five-point Likert scale from 1 (strongly disagree) to 5 (strongly agree). Higher scores represent stronger PE. Prior studies document strong reliability and construct validity across occupations (Zhou and Chen, 2021). For the final PE indicators included in the analysis, Cronbach’s *α* was 0.880.

#### 3.2.3 Innovative work behavior

We measured IWB with the Employee Innovative Behavior Scale by Scott and Bruce (1994). The 6 items capture employees’ innovative behaviors related to idea generation, idea promotion or championing, and idea implementation, for example “I generate creative ideas.” All items were rated on a five-point Likert scale where 1 denotes strongly disagree and 5 denotes strongly agree. Higher values indicate greater innovative behavior. The scale has been widely applied and validated among corporate employees, with consistent evidence for reliability and validity (Liu and Tong, 2022). The IWB construct retained for analysis had a Cronbach’s α of 0.832.

#### 3.2.4 Perceived organizational support

POS was measured using the Survey of Perceived Organizational Support (SPOS-8) developed by Eisenberger et al. (1986). The scale consists of 8 items (e.g., “My organization values my contribution to its well-being”) rated on a five-point Likert scale from 1 (strongly disagree) to 5 (strongly agree). Items 2, 3, 5, and 7 were reverse coded prior to computing the total score, with higher total scores reflecting stronger POS (Bardhoshi et al., 2022; Worley et al., 2009). The SPOS-8 has been validated in studies conducted in the Chinese context and shows strong psychometric properties (Li Y. et al., 2025). Cronbach’s α for the POS indicators used in the final analysis reached 0.930.

### 3.3 Data analysis

We tested the hypotheses using partial least squares structural equation modeling (PLS-SEM) as the primary estimator. The measurement and structural models were estimated in Smart PLS 4.0, and the significance of structural paths was evaluated using bootstrapping with 5,000 resamples. All hypothesis tests, including the structural paths, mediation analysis, moderation analysis, and multi-group analysis, were based on the PLS-SEM model estimated in Smart PLS 4.0. After establishing measurement invariance through the Measurement Invariance of Composite Models (MICOM) procedure, we conducted multi-group analysis (MGA) to probe potential group differences in path coefficients. In addition, confirmatory factor analysis and the common latent factor comparison were conducted as supplementary covariance-based SEM diagnostic checks in IBM SPSS AMOS 26.0. These supplementary checks used the same measurement structure and item set corresponding to the final measurement model reported in Table 2. They were used only to evaluate the measurement structure and possible common-method variance, not to estimate the structural hypotheses.

**Table 2:** Measurement model quality indicators (*N* = 500).

Constructs	Items	Loadings	Cronbach’s α	CR	AVE
TL	TL1	0.853	0.937	0.938	0.761
	TL2	0.854			
	TL3	0.880			
	TL4	0.891			
	TL5	0.876			
	TL6	0.879			
IWB	IWB1	0.722	0.832	0.846	0.541
	IWB2	0.799			
	IWB3	0.766			
	IWB4	0.642			
	IWB5	0.786			
	IWB6	0.685			
PE	PE1	0.701	0.880	0.884	0.543
	PE2	0.721			
	PE4	0.658			
	PE5	0.698			
	PE8	0.773			
	PE9	0.800			
	PE11	0.780			
	PE12	0.753			
POS	POS1	0.843	0.930	0.932	0.670
	POS2	0.849			
	POS3	0.776			
	POS4	0.845			
	POS5	0.800			
	POS6	0.820			
	POS7	0.783			
	POS8	0.830			

CR, composite reliability; AVE, average variance extracted; TL, transformational leadership; PE, psychological empowerment; IWB, innovative work behavior; POS, perceived organizational support. The reliability and validity estimates are based on the final retained indicators shown in the table.

## 4 Results

Before hypothesis testing, data quality and analytical assumptions were checked. Normality was assessed using skewness and kurtosis, with |skewness| < 3 and |kurtosis| < 10 indicating approximate normality (Kim, 2013). Bartlett’s test of sphericity showed that all correlation matrices were suitable for factor analysis (*p* < 0.05; see Table 3).

**Table 3:** Descriptive statistics for the constructs (*N* = 500).

Constructs	N	MIN	MAX	M ± SD	SK	Kur	Bartlett
POS	500	8	40	32.734 ± 6.162	−1.460	3.155	<0.001
PE	500	12	60	27.934 ± 4.356	−1.762	3.745	<0.001
IWB	500	6	30	24.058 ± 4.253	−0.018	−1.323	<0.001
TL	500	8	40	21.960 ± 4.618	−0.440	0.692	<0.001

N, sample size; M ± SD, mean ± standard deviation; MIN, minimum value; MAX, maximum value; SK, skewness; Kur, kurtosis; TL, transformational leadership; PE, psychological empowerment; IWB, innovative work behavior; POS, perceived organizational support; *p*-values < 0.001 indicate that the variables are suitable for factor analysis.

### 4.1 SEM analysis

PLS-SEM was applied to estimate and assess both the measurement and structural components of the research model. The analysis explored the associations between TL and hotel employees’ IWB. The framework comprised four latent constructs represented by 38 indicators, based on responses from 500 participants. Considering the study’s objectives and data characteristics, PLS-SEM was deemed the most suitable analytic strategy (Hair et al., 2021). Accordingly, the hypothesis tests, including the structural paths, mediation analysis, moderation analysis, and multi-group analysis, were estimated in Smart PLS 4.0.

### 4.2 Measurement model

Following Hair et al. (2021), we assessed reliability and validity through the measurement model. For reliability, we examined outer loadings and composite reliability (CR). The overall measurement model reached acceptable reliability and validity standards. For reflective measurement models, an outer loading of 0.708 is generally considered an ideal threshold; however, indicators with lower loadings may still be retained when key criteria such as CR and average variance extracted (AVE) meet the recommended standards and when construct content coverage remains appropriate (Shela et al., 2023). Therefore, this study used an outer loading below 0.60 as the reference standard for item removal and considered item treatment together with CR, AVE, and construct content coverage to avoid mechanical deletion (Ketchen, 2013).

The final item structure was as follows. For TL, the original scale contained 8 items; TL1–TL6 were retained, and TL7 and TL8 were removed. For PE, the original scale contained 12 items; PE1, PE2, PE4, PE5, PE8, PE9, PE11, and PE12 were retained, whereas PE3, PE6, PE7, and PE10 were removed. For IWB, all 6 original items were retained. For POS, all 8 original items were retained. Although IWB4 (0.642), IWB6 (0.685), PE1 (0.701), PE4 (0.658), and PE5 (0.698) were below the ideal threshold of 0.708, each was above the 0.60 removal reference standard. In addition, the corresponding constructs met the recommended reliability and convergent validity criteria, with CR values above 0.70 and AVE values above 0.50. Therefore, these indicators were retained in the final measurement model.

After refinement, the final measurement model retained 6 TL, 8 PE, 6 IWB, and 8 POS items; Table 2 reports Cronbach’s *α*, CR, and AVE for these indicators, with reliability meeting accepted thresholds (α > 0.70; CR > 0.80). Discriminant validity was also assessed using HTMT and the Fornell–Larcker criterion, and the results supported acceptable discriminant validity (see Tables 4, 5).

**Table 4:** Discriminant validity by HTMT (*N* = 500).

Constructs	TL	IWB	PE	POS
TL				
IWB	0.324			
PE	0.670	0.215		
POS	0.618	0.165	0.810	

TL, transformational leadership; PE, psychological empowerment; IWB, innovative work behavior; POS, perceived organizational support.

**Table 5:** Fornell–Larcker criterion matrix (*N* = 500).

Constructs	TL	IWB	PE	POS
TL	***0.872***			
IWB	0.299	***0.735***		
PE	0.598	0.181	***0.748***	
POS	0.578	0.153	0.737	***0.819***

TL, transformational leadership; PE, psychological empowerment; IWB, innovative work behavior; POS, perceived organizational support; the square root of the AVE value is displayed in bold and italic on the diagonal of the table.

### 4.3 Common method bias and supplementary confirmatory factor analysis

An initial assessment of common-method variance used Harman’s single-factor procedure. EFA yielded five factors with eigenvalues above 1; the first explained 35.778%, below the 40% threshold (Podsakoff and Organ, 1986), suggesting that no single factor dominated the covariance among the items. As a supplementary covariance-based SEM diagnostic check, we then specified a common latent factor (CLF) model in IBM SPSS AMOS 26.0. The CLF model and the baseline Confirmatory Factor Analysis (CFA) model used the same measurement structure and item set corresponding to the final measurement model reported in Table 2. The chi-square difference between the baseline model, χ^2^ = 2018.442 (df = 482), and the common latent factor model, χ^2^ = 2019.852 (df = 481), was 1.410 with df = 1 and was not significant (*p* > 0.05). Nevertheless, because the key constructs, including the outcome variable, were assessed through perceptual self-reports in a single survey, residual common-method variance cannot be fully ruled out. We also conducted a supplementary Covariance-Based Structural Equation Modeling (CB-SEM) CFA in IBM SPSS AMOS 26.0 to examine whether the measurement structure was acceptable under covariance-based assumptions. The fit indices were χ^2^/df = 4.188, SRMR = 0.032, GFI = 0.980, NFI = 0.981, IFI = 0.982, TLI = 0.987, CFI = 0.982, and RMSEA = 0.061. Following Hu and Bentler (1998), CFI and TLI greater than 0.90 and RMSEA less than 0.08 indicate acceptable model fit. These CB-SEM fit indices and the CLF comparison are reported only as supplementary diagnostics for measurement validity and common-method variance.

### 4.4 Structural model

#### 4.4.1 Collinearity assessment

We used variance inflation factors (VIF) to check for possible collinearity (Hair et al., 2021). As shown in Table 6, all VIF values were below 3.3. This indicates that collinearity is unlikely to bias the model estimates.

**Table 6:** Assessment of multicollinearity in the structural model (*N* = 500).

Constructs	TL	IWB	PE	POS
TL		1.704	1.526	
IWB				
PE		2.893		
POS		2.541	1.763	

TL, transformational leadership; PE, psychological empowerment; IWB, innovative work behavior; POS, perceived organizational support.

#### 4.4.2 Significance testing of structural paths

Consistent with the primary PLS-SEM estimator, we evaluated the magnitude and significance of structural paths using PLS bootstrapping with 5,000 resamples in Smart PLS 4.0. Following Hair et al. (2021), paths were deemed significant when *t* > 1.96, *p* < 0.05, and the 95% confidence interval excluded zero. All structural paths met these criteria, with full statistics reported in Table 7. For explanatory power, R^2^/adjusted R^2^ values were 0.389/0.382 for IWB and 0.621/0.618 for PE, showing that the predictors explained 38.9 and 62.1% of their variance, respectively. The Q^2^ values for IWB (0.359) and PE (0.419) were both positive, indicating predictive relevance (Table 8).

**Table 7:** Structural model path significance (*N* = 500).

Relationships	Original sample (O)	2.50%	97.50%	*t*	*p*	Results
TL → IWB	0.306	0.211	0.405	6.263	<0.001	Yes
TL → PE	0.227	0.141	0.324	4.880	<0.001	Yes
PE → IWB	0.263	0.125	0.399	3.834	<0.001	Yes

TL, transformational leadership; PE, psychological empowerment; IWB, innovative work behavior.

**Table 8:** Model explanatory power (*N* = 500).

Constructs	*R* ^2^	*R* ^2^_Adjusted_	Q^2^
IWB	0.389	0.382	0.359
PE	0.621	0.618	0.419

PE, psychological empowerment; IWB, innovative work behavior.

#### 4.4.3 Mediation analysis

We applied PLS-SEM to evaluate whether PE functions as a mediator in the relationship between TL and IWB. After controlling for gender, age, and tenure, the analysis indicated a partial mediation, showing that PE accounted for part of the relationship between TL and IWB. Detailed estimates of the indirect effect and relevant statistics are presented in Table 9.

**Table 9:** Mediation effect results (*N* = 500).

Path	Indirect effect	2.50%	97.50%	*t*	*p*	Direct effect	2.50%	97.50%	*t*	*p*	Results
TL → PE → IWB	0.060	0.025	0.104	2.981	0.003	0.306	0.211	0.405	6.263	<0.001	PM

TL, transformational leadership; PE, psychological empowerment; IWB, innovative work behavior; PM, partial mediation.

#### 4.4.4 Moderation analysis

POS moderation was examined by adding interaction terms to the model, with gender, age, and tenure controlled. POS × TL was positively linked to IWB, showing that the TL–IWB link strengthened under higher POS conditions. POS × PE also showed a positive link with IWB, suggesting that PE was more strongly connected to IWB when POS was high. By contrast, POS × TL was not significantly associated with PE, so moderation on this path was not supported.

**Figure 2:**
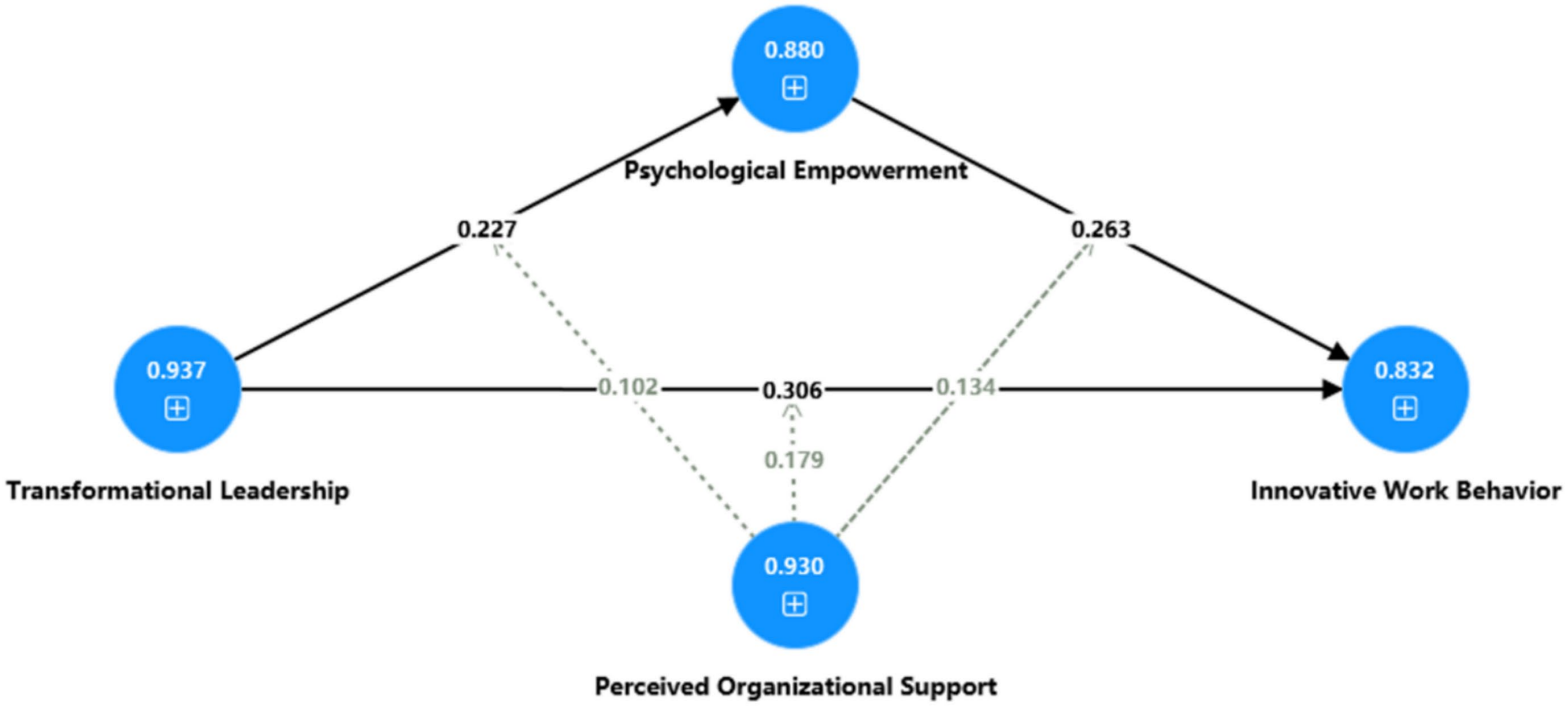
Estimated structural paths in the moderated mediation model. Blue-circle values denote Cronbach’s alpha coefficients; path values denote path coefficients.

A comparison of moderating effects indicates that POS more strongly moderated the TL → IWB path (*β* = 0.179) than the PE → IWB path (*β* = 0.134). These results suggest a more pronounced enhancing role of POS on the direct link between leadership behavior and innovation. Detailed estimates are reported in Table 10, and Figure 2 depicts the tested mediation and moderation paths.

**Table 10:** Moderation effect results (*N* = 500).

Relationships	Original sample (O)	2.50%	97.50%	*t*	*p*	Results
POS × TL → IWB	0.179	0.075	0.290	3.304	<0.001	Supported
POS × TL → PE	0.102	−0.101	0.023	1.409	0.081	Not supported
POS × PE → IWB	0.134	0.067	0.216	3.553	<0.001	Supported

TL, transformational leadership; PE, psychological empowerment; IWB, innovative work behavior; POS, perceived organizational support.

### 4.5 Group-based path comparison

#### 4.5.1 Invariance check across gender groups

Equivalence across gender groups was checked using the MICOM procedure (Henseler et al., 2016). Table 11 shows that all constructs satisfied the criteria for measurement invariance.

**Table 11:** MICOM Step 2: compositional invariance by gender (*N* = 500).

Constructs	Correlation permutation mean	5% Quantile of empirical distribution of *c_u_*	*p*	Compositional invariance?
TL	1.000	1.000	0.685	Yes
IWB	0.991	0.978	0.483	Yes
PE	0.999	0.995	0.620	Yes
POS	1.000	0.999	0.899	Yes

TL, transformational leadership; PE, psychological empowerment; IWB, innovative work behavior; POS, perceived organizational support.

#### 4.5.2 Multi-group comparisons

MGA compared male–female path coefficients using a dummy-coded gender variable (Zhou et al., 2014). As displayed in Table 12, the relationship between TL and PE showed significant gender differences. The path from TL to PE was stronger among female employees than among male employees, with the coefficient for the female group being higher by 0.177.

**Table 12:** Gender-based path estimates (*N* = 500).

Path	Original (Male)	Original (Female)	Difference (Male–Female)	2.50%	97.50%	*p*
TL → PE	0.141	0.318	−0.177	0.211	0.432	0.048

TL, transformational leadership; PE, psychological empowerment.

## 5 Discussion

This study found a significant positive association between TL and hotel employees’ IWB, indicating that stronger TL relates to stronger IWB, which supports H1. Given the cross-sectional and single-source design, this pattern should be interpreted as an association rather than evidence of temporal ordering or a causal relationship. This result aligns with recent evidence. Korku and Kaya (2023) reported that TL encourages employees to challenge routines and provides psychological safety, which is associated with greater innovation intentions and proactivity. Jameel et al. (2025) further showed in tourism and hospitality samples that leader–subordinate relationships grounded in trust and reciprocity correspond to employees’ innovative responses to leadership support. From a leader-member exchange perspective, high-quality exchange relationships, defined by mutual trust, respect, and obligation, are typically associated with greater access to resources, more autonomy, and stronger psychological safety, and these conditions together relate to a more positive innovation orientation. At the same time, divergent findings exist. Aristana et al. (2024) and Sudibjo and Prameswari (2021) reported no significant association between TL and IWB. These discrepancies suggest that contextual features may moderate the TL–IWB link, including industry characteristics, team structures, and cultural orientations. In this context, our observation of a significant positive TL–IWB association within Chinese hotels both echoes the dominant research trend and underscores the need to assess contextual applicability in service organizations with relatively high-power distance and stronger collectivist orientations. Specifically, leaders’ idealized influence, intellectual stimulation, and individualized consideration may relate to greater psychological safety and efficacy among frontline staff, which corresponds to faster conversion of ideas into incremental improvements in service processes and customer experience and, in turn, to stronger IWB (Parveen and Alshehri, 2023). These findings also indicate that future research should examine cultural and organizational variables as boundary conditions in a more systematic manner. Thus, the present result does not simply repeat the established TL–IWB relationship; rather, it situates this relationship within hotel work, where standardized service routines and unpredictable customer demands make leadership-related encouragement particularly relevant to employees’ innovation-oriented responses.

This study also found that TL was significantly positively associated with PE, and PE was significantly positively associated with IWB. Mediation testing further indicated that PE carried part of the association between TL and IWB, supporting H2, H3, and H4. These results align with prior evidence. Abdulrab et al. (2025) reported that vision-based encouragement and individualized consideration from leaders relate to stronger recognition of work value, as well as greater meaning and autonomy among employees. Aristana et al. (2024) likewise documented a positive association between TL and PE. Regarding PE and IWB, Al Daboub et al. (2024) and Sheeba and Christopher (2024) found that higher empowerment corresponds to more proactive exploration, problem identification, and the generation and implementation of innovative ideas. Evidence on the mediating role is also consistent with our findings. Bektas et al. (2025) showed that PE serves as a bridge between leadership behavior and employees’ innovative performance, underscoring the importance of intrinsic motivation in innovation. These patterns accord with the core view of SCT, which holds that behavior reflects ongoing interactions between personal cognitions and environmental conditions. In this study, TL functioned as a key environmental factor. Through vision articulation and individualized support, TL was associated with stronger perceptions of work meaning, competence, and self-determination, which together indicate higher PE. This activated cognitive state, in turn, related closely to employees’ greater tendency to identify problems and try new methods, which are central elements of IWB (Kim and Yoon, 2025). Together, the findings portray a coherent pattern of associations among the contextual factor, psychological process, and behavioral expression. Compared with prior studies that identify PE as a mediator in general organizational or healthcare settings, this study shows that the same cognitive pathway is also relevant in hotel service work, where employees’ sense of meaning, competence, and autonomy corresponds closely to their willingness to engage in idea generation and implementation.

This study further indicates that POS significantly moderates the association between TL and employees’ IWB, which supports H5a. It also strengthens the positive association between PE and IWB, supporting H5c. In contrast, the moderating effect on the association between TL and PE was not significant, so H5b was not supported. A relational interpretation informed by LMX may help explain why POS is associated with a stronger TL–IWB link, but this interpretation should be treated as supplementary because LMX was not measured in the present study. High quality leader–member relationships are commonly discussed as relational contexts linked to employees’ willingness to engage in extra-role behaviors such as innovation (Jelaca et al., 2025). When employees perceive organization wide support and trust, they are more likely to read leaders’ care and encouragement as extensions of the organizational support system rather than as expressions of personal style. This positive organizational attribution consolidates the trust base of leader–member exchange and, consistent with reciprocity norms, relates to employees’ readiness to translate leaders’ innovation expectations into concrete creative actions (Song et al., 2018). From the SCT perspective of environment–cognition–behavior interaction, the moderating effect of POS is particularly salient on the path from PE to IWB (Chouchane et al., 2023). PE reflects employees’ subjective cognitions about efficacy and influence at work and serves as an important internal motivator for innovation (Çetinkaya and Yesilada, 2022). POS, as a critical external resource, provides the safe and well-resourced climate that enables this cognitive state to translate into behavior (Park and Kim, 2022). When employees perceive understanding and tolerance from the organization, they are more likely to enact empowerment-driven innovation intentions, which strengthens the cognition-to-behavior conversion.

Overall, POS primarily serves as a catalyst that helps convert different drivers, whether external leadership behaviors or internal psychological energy, into visible innovation. In the Chinese context, where collectivism and organizational loyalty are emphasized, employees often show strong affective attachment to organizational support and recognition (Lam et al., 2016). In hotel settings characterized by relatively high-power distance, employees may view organizational support as a signal of commitment and trust and therefore respond to leaders’ encouragement with greater innovation. Given the dual pressures of demanding service standards and emotional labor, POS not only eases psychological load but also offers emotional resources and psychological safety that help convert empowerment into observable innovative behavior. This interpretation is consistent with organizational support research, while also extending it by showing that support perceptions may be especially relevant when hotel employees must balance service consistency with flexible responses to customer needs.

Regarding effect size, POS more strongly moderated the relationship between TL and IWB (*β* = 0.179) than the relationship between PE and IWB (*β* = 0.134). This pattern suggests a more pronounced enhancing role of POS on the direct association between leadership behavior and innovation, while its incremental role in converting internal cognitions into behavior appears relatively smaller. One plausible explanation lies in the situational nature of innovation in service organizations. Hotel employees often engage in innovation during task execution and customer interaction. The emotional and resource assurances provided by POS may relate directly to employees’ active responses to leadership expectations, thereby elevating IWB more immediately. In contrast, the effect of PE on behavior typically requires a longer process of internalization and translation and may be less sensitive to immediate contextual cues (Ergun et al., 2025).

Finally, this study found a significant gender difference in the association between TL and PE: the relationship was notably stronger among female employees than among male employees. This result highlights gender as an important moderator within hotel organizations. Female employees’ levels of PE appear to be more sensitive to TL behaviors, which may stem from gender socialization and the resulting differences in emotional responsiveness. Women tend to be more attuned to interpersonal relationships and social support (Khan et al., 2025), and therefore respond more positively to the individualized consideration and inspirational motivation that characterize TL. This leadership style aligns with their expectations for supportive and relationship-oriented leaders and effectively enhances their sense of meaning, autonomy, and impact at work, which represents their PE. In contrast, male employees often focus more on task outcomes and career progression, showing a performance-oriented motivational pattern that makes them less responsive to the emotional and relational aspects of TL. The hospitality industry, which relies heavily on interpersonal interactions and emotional labor, further amplifies this gender difference. Female employees are more likely to interpret TL behaviors as signals of organizational trust and recognition; when leaders express concern for their personal growth and contributions, they tend to develop stronger value identification and affective commitment, which in turn elevates their PE. Male employees, by comparison, may invest less emotional engagement in similar contexts, resulting in a weaker sense of empowerment.

## 6 Implications

### 6.1 Theoretical implications

First, by integrating SCT and LMX, this study developed a comprehensive framework that systematically incorporates leadership behavior, employees’ cognitive processes, and innovation outcomes into a single analytical model. The model advances existing theory by emphasizing the dynamic linkage among environment, cognition, and behavior, demonstrating that TL is not only an external motivational stimulus but also a key contextual factor shaping employees’ internal psychological states. By introducing PE as a central cognitive mechanism, the study strengthens SCT’s proposition that individual cognition serves as a bridge between external contexts and behavioral responses, thereby offering a more integrated theoretical explanation for employees’ IWB.

Second, this study incorporates POS as a moderator into the leadership–employee behavioral mechanism, further clarifying the boundary role of organizational context in employees’ psychological and behavioral processes. By identifying distinct moderating effects across different paths, the study underscores the pivotal function of organizational resources and social support in facilitating the conversion of external leadership behaviors into internal psychological states. This perspective enriches both SCT and LMX by expanding their focus on contextual influences, extending the theoretical understanding of leadership effects from the individual to the organizational level, and highlighting the multilevel interactions underlying the formation of IWB.

Finally, through MGA, this study examined the variability of leadership mechanisms from a gender perspective, offering a new theoretical lens for understanding individual characteristics as moderators of leadership effects. The results indicate that gender, as a socially shaped and stable individual characteristic, may influence how employees perceive and respond to leadership behaviors, leading to variations in the underlying psychological pathways. This finding suggests that future research on leadership and innovation should pay greater attention to the interaction between individual differences and contextual variables, thereby refining theoretical models of leadership effectiveness across different employee groups.

### 6.2 Practical implications

For hotel managers, this study highlights the central role of leadership behaviors in encouraging employees’ IWB. Managers may strengthen characteristics of TL by setting challenging goals, offering timely feedback, and providing individualized guidance to help employees develop a sense of meaning and self-efficacy at work. Authorizing decision latitude and conveying trust can enhance PE, giving employees greater autonomy and influence in their roles, which relates to the generation and enactment of innovative ideas. Managers should also attend to employees’ emotional and developmental needs and create an open and inclusive communication climate where employees feel supported to proactively refine service processes.

At the organizational level, the findings point to POS as a critical resource that can reinforce leadership effects. Hotel organizations can establish systematic support mechanisms, including training programs, innovation-oriented incentives, and clear feedback channels, to strengthen employees’ feelings of trust and psychological safety. By embedding a supportive culture and learning routines in daily management, organizations can reduce the perceived risk of innovation and nurture stronger identification with the organization. It is also advisable to monitor PE and innovation-related performance across different employee groups and to provide targeted resources and development opportunities, especially for female staff and frontline roles, to sustain a balanced internal climate for innovation.

At the industry and societal levels, this work suggests practical routes for strengthening innovation capacity and service quality in hospitality. As the service economy grows, innovation functions as a core driver of high-quality development. Industry associations and policy makers can prioritize the cultivation of TL and PE through professional training and policy support that build innovation awareness and psychological capital. Encouraging experience sharing and collaboration among firms can foster an industry culture characterized by trust and support, which is associated with higher employee well-being and better customer experience and contributes to the sustainable development of the hotel sector.

## 7 Study limitations and directions for future research

Although the study modeled mediated and moderated links among TL, PE, and IWB and considered the roles of POS and gender, several limitations remain. First, the cross-sectional questionnaire design offers limited insight into temporal dynamics among the variables. Because TL, PE, POS, and IWB were measured at a single time point, the observed paths should be interpreted as associations within the proposed model rather than as evidence of temporal ordering or directional relationships. This design also limits the ability to rule out alternative patterns, such as the possibility that employees reporting higher IWB may also report more favorable perceptions of TL, PE, or POS. Future work can employ longitudinal tracking, time-lagged questionnaire designs, or situational experiments to examine trajectories of PE and IWB under different leadership styles, which would clarify the temporal sequence among the constructs with greater precision. Second, the data primarily rely on self-reports, which may reflect social desirability and common-source bias. Although the study included statistical checks for common method bias, these procedures cannot fully address response-style bias, consistency motives, positive affectivity, or respondents’ tendency to present themselves favorably. Accordingly, the associations among TL, PE, POS, and IWB may be overstated. Subsequent studies can incorporate multi-source information, such as supervisor-rated IWB, peer evaluations, objective performance indicators, behavioral observations, and a theoretically unrelated marker variable, to provide a more objective basis for evaluating the associations among TL, PE, POS, and IWB. Third, the present study centers on intraorganizational mechanisms and gives limited attention to contextual factors such as industry culture and organizational structure. Future research can incorporate cultural differences, hierarchical levels, and team climate into the model to test boundary effects across contexts and to refine theoretical accounts of the leadership–innovation linkage. Fourth, although the sample covered participating hotels with different regional locations, service levels, and ownership structures, the analysis was conducted at the individual level. The present PLS-SEM model did not explicitly account for hotel-level clustering or hotel-level random parameters. Therefore, the statistical inference should be interpreted with caution, and future research could retain more detailed hotel-level sampling information and use multilevel or cluster-robust approaches to examine whether the observed individual-level associations are consistent across hotels.

## 8 Conclusion

Guided by an integrated lens of SCT and LMX, this study systematically evaluated the mechanisms connecting TL with hotel employees’ IWB by testing the mediating role of PE and the moderating role of POS. The results indicate that TL shows a positive association with IWB. PE partially mediated the association between TL and IWB. POS positively moderated both the TL to IWB association and the PE to IWB association. Furthermore, the MGA results showed that the TL–PE linkage was more pronounced in the female subgroup than in the male subgroup. Taken together, the findings portray a synergistic pattern among leadership behavior, PE, and organizational support, extend the application of SCT and LMX to hotel settings, and offer theoretical as well as empirical bases for promoting employees’ innovative behavior and informing organizational management practices.

## Data Availability

The datasets presented in this study can be found in online repositories. The names of the repository/repositories and accession number(s) can be found in the article/supplementary material.
